# Venlafaxine besylate monohydrate

**DOI:** 10.1107/S1600536813027542

**Published:** 2013-11-06

**Authors:** Carolina H. Corvalan, Daniel R. Vega

**Affiliations:** a Gerencia Materiales, GAEN – CAC – CNEA, Av. Gral. Paz 1499, (1650) San Martín, Buenos Aires, Argentina; b Departamento Física de la Materia Condensada, Gerencia Investigación y Aplicaciones, GAIyANN – CAC – CNEA, Av. Gral. Paz 1499, (1650) San Martín, Buenos Aires, Argentina; c Escuela de Ciencia y Tecnología, Campus Miguelete, Edificio Tornavías, UNSAM, Martín de Irigoyen N 3100, (1650) San Martín - Buenos Aires, Argentina

## Abstract

The title compound {systematic name: [2-(1-hydroxycyclohexyl)-2-(4-methoxyphenyl)ethyl]dimethylazanium benzene­sulfonate monohydrate}, C_17_H_28_NO_2_^+^·C_6_H_5_O_3_S^−^·H_2_O, is a besylate salt hydrate of the anti­depressant drug venlafaxine. In the crystal, besylate anions and water mol­ecules self-assemble, forming hydrogen-bonded dimers linked around inversion centers, with graph set *R*_4_^4^(6). The crystal packing features a chain of alternate dimers and venlafaxine cations in the *b*-axis direction with the components linked by O—H⋯O hydrogen bonds and C—H⋯O and C—H⋯π inter­actions. This is the first example of a venlafaxine cation with a closed conformation, as it features an intra­molecular N—H⋯O inter­action involving the protonated N atom.

## Related literature

For background information, see: Venu *et al.* (2008 ▶); Tessler & Goldberg (2004 ▶); Van Eupen *et al.* (2008 ▶); Yardley *et al.* (1990 ▶); Banjeree *et al.* (2005 ▶); Vega *et al.* (2000 ▶); Sivalakshmidevi *et al.* (2002 ▶); Roy *et al.* (2007 ▶). For ring-puckering calculations, see: Cremer & Pople (1975 ▶). For graph-set notation, see: Bernstein *et al.* (1995 ▶). For a description of the Cambridge Structural Database, see: Allen (2002 ▶). 
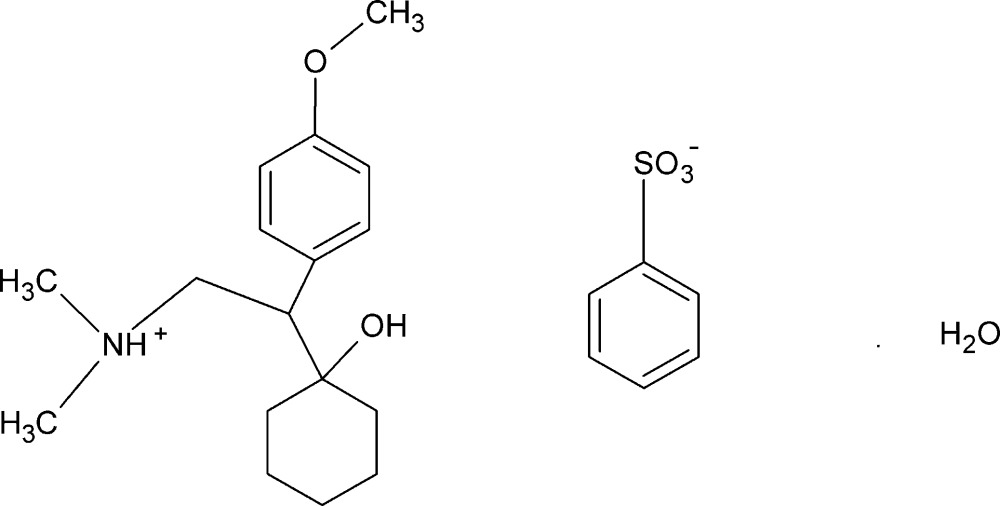


## Experimental

### 

#### Crystal data

C_17_H_28_NO_2_^+^·C_6_H_5_O_3_S^−^·H_2_O
*M* *_r_* = 453.58
Triclinic, 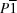

*a* = 10.1163 (5) Å
*b* = 10.2176 (4) Å
*c* = 13.8162 (6) Å
α = 72.074 (4)°
β = 70.108 (4)°
γ = 63.889 (5)°
*V* = 1184.53 (11) Å^3^
*Z* = 2
Mo *K*α radiation
μ = 0.17 mm^−1^
*T* = 293 K
0.70 × 0.30 × 0.10 mm

#### Data collection

Agilent Xcalibur (Eos, Gemini) diffractometer
Absorption correction: multi-scan (*CrysAlis PRO*; Agilent, 2010 ▶) *T*_min_ = 0.842, *T*_max_ = 1.000
10063 measured reflections
5366 independent reflections
3072 reflections with *I* > 2σ(*I*)
*R*_int_ = 0.020

#### Refinement

*R*[*F* ^2^ > 2σ(*F* ^2^)] = 0.053
*wR*(*F* ^2^) = 0.163
*S* = 1.02
5366 reflections
292 parameters
H atoms treated by a mixture of independent and constrained refinement
Δρ_max_ = 0.54 e Å^−3^
Δρ_min_ = −0.44 e Å^−3^

### 

Data collection: *CrysAlis PRO* (Agilent, 2010 ▶); cell refinement: *CrysAlis PRO*; data reduction: *CrysAlis PRO*; program(s) used to solve structure: *SHELXS97* (Sheldrick, 2008 ▶); program(s) used to refine structure: *SHELXL97* (Sheldrick, 2008 ▶); molecular graphics: *ORTEP-3 for Windows* (Farrugia, 2012 ▶); software used to prepare material for publication: *WinGX* (Farrugia, 2012 ▶) and *PLATON* (Spek, 2009 ▶).

## Supplementary Material

Crystal structure: contains datablock(s) global, I. DOI: 10.1107/S1600536813027542/gg2130sup1.cif

Structure factors: contains datablock(s) I. DOI: 10.1107/S1600536813027542/gg2130Isup2.hkl

Supplementary material file. DOI: 10.1107/S1600536813027542/gg2130Isup3.cml

Additional supplementary materials:  crystallographic information; 3D view; checkCIF report

## Figures and Tables

**Table 1:** Hydrogen-bond geometry (Å, °) *Cg*1 is the centroid of the C1–C6 ring.

*D*—H⋯*A*	*D*—H	H⋯*A*	*D*⋯*A*	*D*—H⋯*A*
O2—H2*A*⋯O1*W*	0.81 (3)	1.87 (3)	2.673 (3)	173 (3)
O1*W*—H1*WB*⋯O3*B*	0.83 (3)	1.92 (3)	2.711 (4)	159 (3)
O1*W*—H1*WA*⋯O2*B* ^i^	0.88 (3)	1.91 (3)	2.785 (4)	175 (3)
N1—H1⋯O2	0.78 (3)	2.05 (3)	2.719 (2)	143 (3)
C15—H15*C*⋯O2*B*	0.96	2.68	3.468 (4)	140
C16—H16*A*⋯O1*B* ^ii^	0.96	2.44	3.395 (4)	172
C2—H2⋯*Cg*1	0.93	3.17	3.928	140

Symmetry codes: (i) 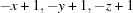; (ii) 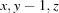.

**Fig. 1.**
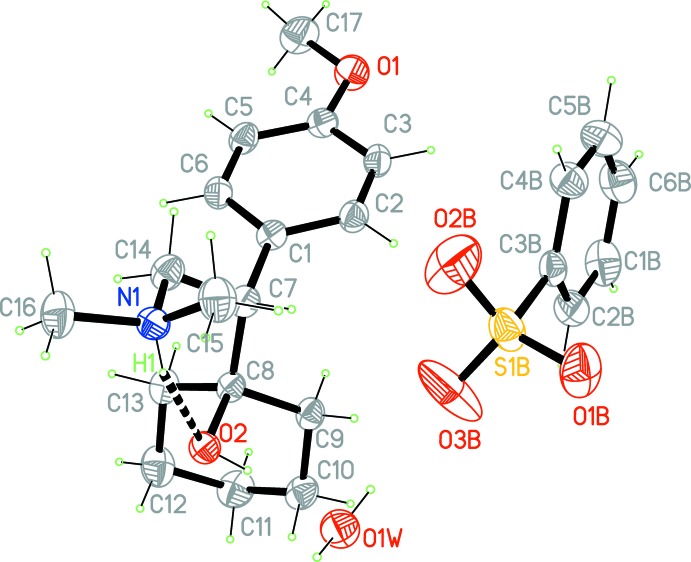
A view of (I). Displacement ellipsoids are drawn at the 30% probability level. Intramolecular hydrogen bond N1—H1···O2 is drawn as dashed line.

**Fig. 2.**
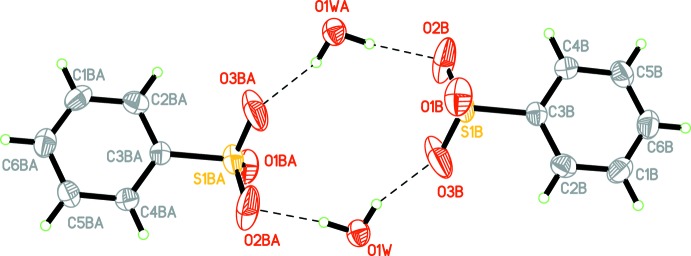
Besylate anions and water molecules around an inversion center, determining a dimmer-like hydrogen-bonded.

**Fig. 3.**
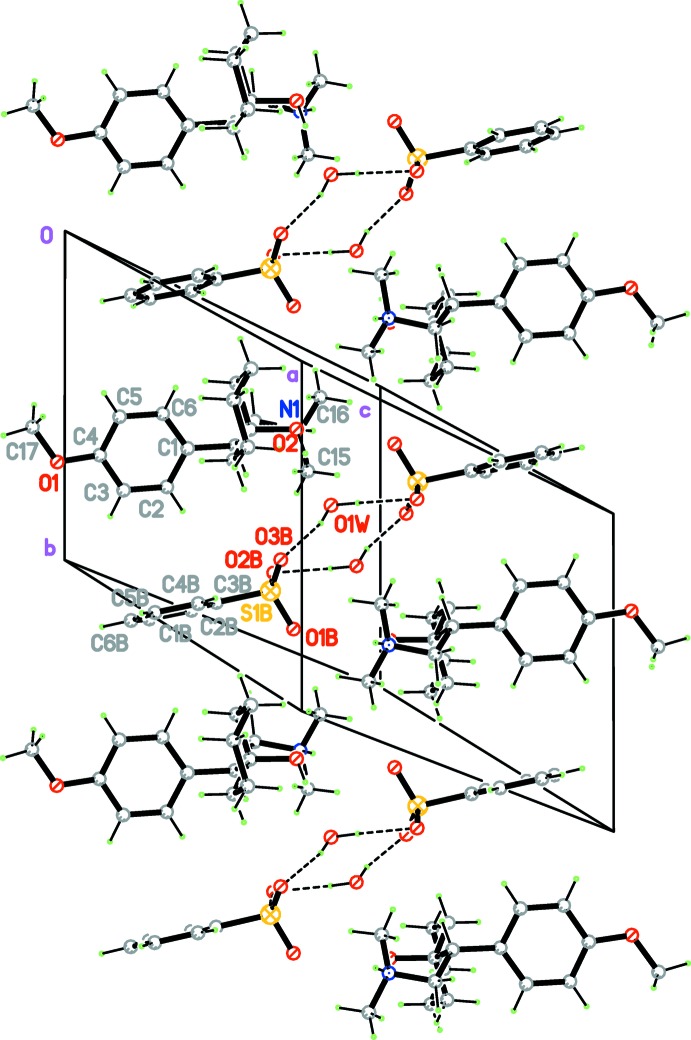
Alternate dimmers and venlafaxine cations determining a chain running along c axis.

## supplementary crystallographic information

### 1. Comment

The title compound (I) is a monohydrate of the 1:1 salt of 1-[2-dimethylamino-1-(4-methoxyphenyl)-ethyl]cyclohexanol with benzenesulphonic acid. Venlafaxine besylate is an antidepressant drug belonging to the class of serotonin norepinephrine reuptake inhibitors. The asymmetric unit consists of a venalfaxine cation, a besylate anion and a water molecule (Fig. 1).

The dimethylaminomethyl group of the venlafaxine cation is protonated by besylic acid. The hydroxy group lies in an axial position with respect to the cyclohexane ring and C7 is located at an equatorial position. Inspecting CSD (Allen, 2002), different conformations could be found for Venlafaxine. An open conformation –T shaped geometry- (Venu *et al.*, 2008), with the dimethylaminomethyl and cyclohexanol groups representing the arms, is observed when venlafaxine is protonated (cation), whereas a closed conformation can be seen when venlafaxine is a free base. The closed conformation observed in the free base molecule is mainly due to the presence of an intra-molecular hydrogen bond provided by O2—H2···N1, then the torsion angle C8—C7—C14—N1 assumes a value that allows the approaching of H2 to N1. The mean value of the torsion angle C8—C7—C14—N1 obtained on seven free molecules is 62 (1)° (refcodes OCALAG, two molecules in OCALAG01, OCALAG02, YISFOW, YISFOW01 and YISFOW02) (Tessler & Goldberg, 2004, Van Eupen *et al.*, 2008). This intramolecular hydrogen bond is absent in the Venlafaxine cation, probably due to the presence of an extra hydrogen atom at N1. The mean value of the torsion angle C8—C7—C14—N1 on six cation molecules is 138 (7)° (refcodes KIDGUZ, VAWQAM, WOBMUV, WOBMUV01 and two molecules in WOBMUV02) (Yardley *et al.*, 1990, Banjeree *et al.*, 2005, Vega *et al.*, 2000, Sivalakshmidevi *et al.*, 2002, Roy *et al.*, 2007), featuring the open conformation for cations. However, the crystal structure introduced here is the first case where a Venlafaxine cation is in a closed conformation. The torsion angle C8—C7—C14—N1 is 71.9 (2)°. Although the closed conformation is provided by an intramolecular hydrogen bond, in this opportunity a different hydrogen bond is present because it is provided by the protonated nitrogen atom N1, where O2 acts as an acceptor (Fig 1)

The cyclohexyl ring assumes a chair conformation, with puckering amplitude Q_T_=0.564 Å, τ=0.67° and φ=262.3° (Cremer & Pople, 1975), where C8 and C11 are located at 0.666 (3) Å from the C9/C10/C12/C13 mean plane. On average, the endocyclic bonds have angles close to the tetrahedral value [110.7 (8)°] and torsion angles of 55.3 (4)°.

Besylate anions and water molecules are self-assembled to determine a dimer-like hydrogen-bonded link around an inversion center. Two centrosymmetric water molecules are bonded to a couple of also centrosymmetric besylate anions (Fig 2). Each water molecule acts as a donor of two hydrogen bonds determining a graph set *R*_4_^4^(6) in the dimer.

Venlafaxine cation is located between two b-translated dimers, generating several interactions. On one side, O2—H2A···O1W, C15—H15C···O2B and C2—H2···CG1 join a Venlafaxine cation to the water molecule and the besylate anion of one of the dimmers. On the other side, C16—H16A···O1B(*x*,*y* - 1,*z*) and C6—H6···CG1(*x*,*y* - 1,*z*) link a Venlafaxine cation to the besylate anion belonging to the other dimmer (CG1 is defined as the centroid of the ring C1, C2, C3, C4, C5 and C6 atoms). These interactions, some very weak in character, give cohesion to the crystal packing, determining a chain of alternate dimmers and venlafaxine cations along the *b* axis.

The water molecules are held in the crystal structure in a way that desolvation occurs as an isolated event. A differential scanning calorimetry trace shows a single endotherm with a mid-point temperature of around 387 (2) K (heating rate 10 K min^-1^). The water molecule is involved in the formation of as many as three strong hydrogen bonds, bridging the besylate and venlafaxine ions (Fig. 2 and Table 1).

### 2. Refinement

The O atoms in the besylate anion exhibited some disorder, with *U*_eq_ values for O2B and O3B large in relation to the rest of the molecule, thus giving larger *U*_eq_(max)/*U*_eq_(min) ratios. Attempts to refine a split model of these atoms gave no better results. The H atoms were refined using a riding model while keeping their isotropic displacement parameters constrained to 1.2 (H attached to aromatic, methine and methylene C atoms) and 1.5 (H atoms attached to methyl C and N) times larger than those of their carrier atoms. The H atoms in water molecule were refined keeping their isotropic displacement parameters constrained to 1.4 times larger than the O carrier atom. H1 and H2A were found in the Fourier Difference Map and refined keeping their isotropic displacement parameters constrained to 1.2 and 1.5 times larger than the carrier atoms respectively. Data from two different single crystals were collected and similar positions were obtained for H1 and H2A atoms.

### Crystal data

**Table:**

C_17_H_28_NO_2_^+^·C_6_H_5_O_3_S^−^·H_2_O	*Z* = 2
*M**_r_* = 453.58	*F*(000) = 488
Triclinic, *P*1	*D*_x_ = 1.272 Mg m^−^^3^
Hall symbol: -P 1	Mo *K*α radiation, λ = 0.71073 Å
*a* = 10.1163 (5) Å	Cell parameters from 3716 reflections
*b* = 10.2176 (4) Å	θ = 3.8–28.8°
*c* = 13.8162 (6) Å	µ = 0.17 mm^−^^1^
α = 72.074 (4)°	*T* = 293 K
β = 70.108 (4)°	Prism, colourless
γ = 63.889 (5)°	0.70 × 0.30 × 0.10 mm
*V* = 1184.53 (11) Å^3^	

### Data collection

**Table:**

Agilent Xcalibur (Eos, Gemini) diffractometer	5366 independent reflections
Radiation source: Enhance (Mo) X-ray Source	3072 reflections with *I* > 2σ(*I*)
Graphite monochromator	*R*_int_ = 0.020
Detector resolution: 16.1158 pixels mm^-1^	θ_max_ = 28.8°, θ_min_ = 3.8°
ω scans	*h* = −13→12
Absorption correction: multi-scan (*CrysAlis PRO*; Agilent, 2010)	*k* = −12→13
*T*_min_ = 0.842, *T*_max_ = 1.000	*l* = −17→17
10063 measured reflections	

### Refinement

**Table:**

Refinement on *F*^2^	Primary atom site location: structure-invariant direct methods
Least-squares matrix: full	Secondary atom site location: difference Fourier map
*R*[*F*^2^ > 2σ(*F*^2^)] = 0.053	Hydrogen site location: mixed
*wR*(*F*^2^) = 0.163	H atoms treated by a mixture of independent and constrained refinement
*S* = 1.02	*w* = 1/[σ^2^(*F*_o_^2^) + (0.0896*P*)^2^] where *P* = (*F*_o_^2^ + 2*F*_c_^2^)/3
5366 reflections	(Δ/σ)_max_ < 0.001
292 parameters	Δρ_max_ = 0.54 e Å^−^^3^
0 restraints	Δρ_min_ = −0.44 e Å^−^^3^

### Special details

**Table:**

Geometry. All e.s.d.'s (except the e.s.d. in the dihedral angle between two l.s. planes) are estimated using the full covariance matrix. The cell e.s.d.'s are taken into account individually in the estimation of e.s.d.'s in distances, angles and torsion angles; correlations between e.s.d.'s in cell parameters are only used when they are defined by crystal symmetry. An approximate (isotropic) treatment of cell e.s.d.'s is used for estimating e.s.d.'s involving l.s. planes.
Refinement. Refinement of *F*^2^ against ALL reflections. The weighted *R*-factor *wR* and goodness of fit *S* are based on *F*^2^, conventional *R*-factors *R* are based on *F*, with *F* set to zero for negative *F*^2^. The threshold expression of *F*^2^ > σ(*F*^2^) is used only for calculating *R*-factors(gt) *etc*. and is not relevant to the choice of reflections for refinement. *R*-factors based on *F*^2^ are statistically about twice as large as those based on *F*, and *R*- factors based on ALL data will be even larger.

### Fractional atomic coordinates and isotropic or equivalent isotropic displacement parameters (Å^2^)

**Table:**

	*x*	*y*	*z*	*U*_iso_*/*U*_eq_	
C1	0.1498 (2)	0.4789 (2)	0.26437 (16)	0.0404 (5)	
C2	0.1147 (3)	0.6298 (2)	0.22570 (18)	0.0460 (5)	
H2	0.1656	0.6771	0.2385	0.055*	
C3	0.0063 (3)	0.7108 (2)	0.16894 (18)	0.0484 (6)	
H3	−0.0147	0.8116	0.1436	0.058*	
C4	−0.0715 (3)	0.6434 (2)	0.14941 (17)	0.0447 (5)	
C5	−0.0402 (3)	0.4941 (2)	0.18763 (18)	0.0479 (6)	
H5	−0.0923	0.4476	0.1752	0.057*	
C6	0.0686 (3)	0.4143 (2)	0.24438 (18)	0.0471 (6)	
H6	0.0885	0.3137	0.2702	0.057*	
C7	0.2715 (3)	0.3927 (2)	0.32531 (16)	0.0424 (5)	
H7	0.2842	0.4664	0.3493	0.051*	
C8	0.4301 (2)	0.3089 (2)	0.25829 (15)	0.0381 (5)	
C9	0.4845 (3)	0.4202 (2)	0.16915 (16)	0.0429 (5)	
H9A	0.4120	0.4721	0.1257	0.051*	
H9B	0.4879	0.4931	0.1987	0.051*	
C10	0.6391 (3)	0.3478 (3)	0.10163 (18)	0.0533 (6)	
H10A	0.7138	0.3052	0.1431	0.064*	
H10B	0.6655	0.4224	0.0446	0.064*	
C11	0.6436 (3)	0.2272 (3)	0.05707 (19)	0.0633 (7)	
H11A	0.7460	0.1782	0.0184	0.076*	
H11B	0.5777	0.2710	0.0090	0.076*	
C12	0.5921 (3)	0.1142 (3)	0.1451 (2)	0.0644 (7)	
H12A	0.6645	0.0630	0.1886	0.077*	
H12B	0.5896	0.0411	0.1153	0.077*	
C13	0.4353 (3)	0.1876 (2)	0.21283 (18)	0.0506 (6)	
H13A	0.4083	0.1130	0.2697	0.061*	
H13B	0.3612	0.2304	0.1709	0.061*	
C14	0.2189 (3)	0.2928 (3)	0.42343 (17)	0.0492 (6)	
H14A	0.1121	0.3436	0.4529	0.059*	
H14B	0.2303	0.2038	0.4046	0.059*	
C15	0.2630 (3)	0.3696 (3)	0.5604 (2)	0.0605 (7)	
H15A	0.3226	0.3349	0.6109	0.073*	
H15B	0.1574	0.3994	0.5955	0.073*	
H15C	0.2817	0.4528	0.5108	0.073*	
C16	0.2888 (4)	0.1159 (3)	0.5811 (2)	0.0655 (7)	
H16A	0.3171	0.0381	0.5443	0.079*	
H16B	0.1854	0.1393	0.6206	0.079*	
H16C	0.3535	0.0837	0.6279	0.079*	
C17	−0.2610 (3)	0.6691 (3)	0.0731 (2)	0.0761 (9)	
H17A	−0.3315	0.7430	0.0331	0.091*	
H17B	−0.3155	0.6286	0.1384	0.091*	
H17C	−0.1928	0.5913	0.0344	0.091*	
N1	0.3050 (2)	0.2494 (2)	0.50483 (14)	0.0416 (5)	
H1	0.390 (3)	0.232 (3)	0.4746 (19)	0.050*	
O1	−0.1776 (2)	0.73441 (18)	0.09278 (14)	0.0630 (5)	
O2	0.53164 (17)	0.23490 (16)	0.32729 (11)	0.0431 (4)	
H2A	0.569 (3)	0.291 (3)	0.326 (2)	0.065*	
S1B	0.31436 (9)	0.78902 (7)	0.40428 (6)	0.0623 (2)	
C1B	0.2016 (4)	1.0171 (3)	0.1316 (2)	0.0693 (8)	
H1B	0.2502	1.0263	0.0607	0.083*	
C2B	0.2834 (3)	0.9237 (3)	0.2047 (2)	0.0603 (7)	
H2B	0.3869	0.8709	0.1833	0.072*	
C3B	0.2115 (3)	0.9089 (2)	0.30976 (18)	0.0436 (5)	
C4B	0.0585 (3)	0.9889 (3)	0.3397 (2)	0.0551 (6)	
H4B	0.0086	0.9808	0.4103	0.066*	
C5B	−0.0207 (3)	1.0810 (3)	0.2649 (2)	0.0671 (7)	
H5B	−0.1244	1.1339	0.2853	0.080*	
C6B	0.0514 (4)	1.0953 (3)	0.1618 (2)	0.0675 (8)	
H6B	−0.0027	1.1590	0.1119	0.081*	
O1B	0.3786 (3)	0.8685 (2)	0.42970 (16)	0.0827 (7)	
O2B	0.2046 (3)	0.7435 (3)	0.4929 (2)	0.1248 (11)	
O3B	0.4211 (3)	0.6657 (3)	0.3590 (3)	0.1424 (13)	
O1W	0.6618 (2)	0.4063 (2)	0.33801 (15)	0.0604 (5)	
H1WA	0.700 (4)	0.357 (3)	0.393 (2)	0.085*	
H1WB	0.590 (4)	0.480 (3)	0.360 (2)	0.085*	

### Atomic displacement parameters (Å^2^)

**Table:**

	*U*^11^	*U*^22^	*U*^33^	*U*^12^	*U*^13^	*U*^23^
C1	0.0412 (13)	0.0455 (12)	0.0362 (11)	−0.0181 (10)	−0.0109 (10)	−0.0053 (9)
C2	0.0488 (14)	0.0440 (12)	0.0520 (13)	−0.0219 (11)	−0.0156 (11)	−0.0070 (10)
C3	0.0525 (15)	0.0387 (11)	0.0544 (14)	−0.0202 (11)	−0.0157 (12)	−0.0012 (10)
C4	0.0434 (13)	0.0500 (12)	0.0389 (12)	−0.0179 (10)	−0.0133 (10)	−0.0012 (9)
C5	0.0507 (14)	0.0521 (13)	0.0517 (13)	−0.0275 (11)	−0.0170 (11)	−0.0062 (10)
C6	0.0497 (14)	0.0398 (11)	0.0534 (14)	−0.0194 (11)	−0.0160 (11)	−0.0028 (10)
C7	0.0465 (14)	0.0450 (11)	0.0388 (12)	−0.0200 (10)	−0.0124 (10)	−0.0050 (9)
C8	0.0421 (13)	0.0422 (11)	0.0321 (10)	−0.0192 (10)	−0.0123 (9)	−0.0010 (8)
C9	0.0520 (14)	0.0431 (11)	0.0368 (11)	−0.0229 (10)	−0.0174 (10)	0.0035 (9)
C10	0.0562 (16)	0.0611 (14)	0.0407 (13)	−0.0298 (13)	−0.0085 (11)	0.0013 (11)
C11	0.0676 (19)	0.0750 (17)	0.0441 (14)	−0.0310 (14)	0.0027 (13)	−0.0185 (12)
C12	0.083 (2)	0.0551 (14)	0.0554 (16)	−0.0262 (14)	−0.0063 (14)	−0.0210 (12)
C13	0.0628 (16)	0.0480 (12)	0.0462 (13)	−0.0279 (12)	−0.0119 (12)	−0.0061 (10)
C14	0.0546 (15)	0.0547 (13)	0.0428 (13)	−0.0269 (12)	−0.0136 (11)	−0.0029 (10)
C15	0.0750 (19)	0.0539 (14)	0.0577 (15)	−0.0256 (13)	−0.0152 (14)	−0.0155 (12)
C16	0.084 (2)	0.0528 (14)	0.0547 (15)	−0.0323 (14)	−0.0156 (14)	0.0054 (12)
C17	0.069 (2)	0.089 (2)	0.083 (2)	−0.0360 (17)	−0.0439 (17)	0.0046 (16)
N1	0.0405 (11)	0.0428 (10)	0.0328 (10)	−0.0127 (9)	−0.0055 (8)	−0.0034 (7)
O1	0.0619 (11)	0.0625 (10)	0.0681 (11)	−0.0232 (9)	−0.0367 (9)	0.0068 (8)
O2	0.0455 (9)	0.0439 (8)	0.0391 (8)	−0.0175 (7)	−0.0177 (7)	0.0030 (6)
S1B	0.0732 (5)	0.0442 (3)	0.0774 (5)	−0.0137 (3)	−0.0421 (4)	−0.0073 (3)
C1B	0.099 (2)	0.0623 (16)	0.0455 (15)	−0.0356 (17)	−0.0123 (15)	−0.0067 (12)
C2B	0.0573 (17)	0.0531 (14)	0.0662 (18)	−0.0184 (12)	−0.0067 (14)	−0.0182 (13)
C3B	0.0537 (15)	0.0333 (10)	0.0514 (14)	−0.0177 (10)	−0.0181 (11)	−0.0098 (9)
C4B	0.0549 (16)	0.0556 (14)	0.0499 (14)	−0.0169 (12)	−0.0096 (12)	−0.0124 (11)
C5B	0.0560 (17)	0.0624 (16)	0.074 (2)	−0.0098 (13)	−0.0244 (15)	−0.0103 (14)
C6B	0.083 (2)	0.0570 (15)	0.0635 (18)	−0.0200 (15)	−0.0343 (16)	−0.0039 (13)
O1B	0.1121 (18)	0.0715 (12)	0.0969 (16)	−0.0428 (12)	−0.0610 (14)	−0.0056 (11)
O2B	0.127 (2)	0.136 (2)	0.1152 (19)	−0.0817 (19)	−0.0755 (18)	0.0723 (17)
O3B	0.154 (3)	0.0835 (15)	0.183 (3)	0.0502 (15)	−0.122 (2)	−0.0687 (17)
O1W	0.0624 (13)	0.0595 (11)	0.0675 (12)	−0.0208 (9)	−0.0275 (10)	−0.0124 (9)

### Geometric parameters (Å, º)

**Table:**

C1—C2	1.388 (3)	C14—H14A	0.9700
C1—C6	1.390 (3)	C14—H14B	0.9700
C1—C7	1.515 (3)	C15—N1	1.476 (3)
C2—C3	1.375 (3)	C15—H15A	0.9600
C2—H2	0.9300	C15—H15B	0.9600
C3—C4	1.378 (3)	C15—H15C	0.9600
C3—H3	0.9300	C16—N1	1.480 (3)
C4—O1	1.369 (2)	C16—H16A	0.9600
C4—C5	1.378 (3)	C16—H16B	0.9600
C5—C6	1.377 (3)	C16—H16C	0.9600
C5—H5	0.9300	C17—O1	1.412 (3)
C6—H6	0.9300	C17—H17A	0.9600
C7—C14	1.516 (3)	C17—H17B	0.9600
C7—C8	1.559 (3)	C17—H17C	0.9600
C7—H7	0.9800	N1—H1	0.78 (3)
C8—O2	1.441 (2)	O2—H2A	0.81 (2)
C8—C13	1.527 (3)	S1B—O3B	1.405 (2)
C8—C9	1.530 (3)	S1B—O1B	1.4150 (18)
C9—C10	1.509 (3)	S1B—O2B	1.459 (3)
C9—H9A	0.9700	S1B—C3B	1.762 (2)
C9—H9B	0.9700	C1B—C6B	1.352 (4)
C10—C11	1.516 (3)	C1B—C2B	1.378 (4)
C10—H10A	0.9700	C1B—H1B	0.9300
C10—H10B	0.9700	C2B—C3B	1.379 (3)
C11—C12	1.523 (3)	C2B—H2B	0.9300
C11—H11A	0.9700	C3B—C4B	1.377 (3)
C11—H11B	0.9700	C4B—C5B	1.377 (3)
C12—C13	1.526 (4)	C4B—H4B	0.9300
C12—H12A	0.9700	C5B—C6B	1.357 (4)
C12—H12B	0.9700	C5B—H5B	0.9300
C13—H13A	0.9700	C6B—H6B	0.9300
C13—H13B	0.9700	O1W—H1WA	0.88 (3)
C14—N1	1.496 (3)	O1W—H1WB	0.84 (3)
			
C2—C1—C6	116.87 (19)	H13A—C13—H13B	107.9
C2—C1—C7	120.05 (18)	N1—C14—C7	113.01 (18)
C6—C1—C7	123.08 (18)	N1—C14—H14A	109.0
C3—C2—C1	121.5 (2)	C7—C14—H14A	109.0
C3—C2—H2	119.2	N1—C14—H14B	109.0
C1—C2—H2	119.2	C7—C14—H14B	109.0
C2—C3—C4	120.4 (2)	H14A—C14—H14B	107.8
C2—C3—H3	119.8	N1—C15—H15A	109.5
C4—C3—H3	119.8	N1—C15—H15B	109.5
O1—C4—C5	124.9 (2)	H15A—C15—H15B	109.5
O1—C4—C3	115.6 (2)	N1—C15—H15C	109.5
C5—C4—C3	119.5 (2)	H15A—C15—H15C	109.5
C6—C5—C4	119.51 (19)	H15B—C15—H15C	109.5
C6—C5—H5	120.2	N1—C16—H16A	109.5
C4—C5—H5	120.2	N1—C16—H16B	109.5
C5—C6—C1	122.22 (19)	H16A—C16—H16B	109.5
C5—C6—H6	118.9	N1—C16—H16C	109.5
C1—C6—H6	118.9	H16A—C16—H16C	109.5
C1—C7—C14	110.96 (18)	H16B—C16—H16C	109.5
C1—C7—C8	114.09 (16)	O1—C17—H17A	109.5
C14—C7—C8	112.73 (18)	O1—C17—H17B	109.5
C1—C7—H7	106.1	H17A—C17—H17B	109.5
C14—C7—H7	106.1	O1—C17—H17C	109.5
C8—C7—H7	106.1	H17A—C17—H17C	109.5
O2—C8—C13	105.98 (16)	H17B—C17—H17C	109.5
O2—C8—C9	110.41 (16)	C15—N1—C16	110.07 (18)
C13—C8—C9	109.26 (17)	C15—N1—C14	113.67 (18)
O2—C8—C7	106.88 (15)	C16—N1—C14	110.92 (19)
C13—C8—C7	114.63 (18)	C15—N1—H1	105.9 (18)
C9—C8—C7	109.58 (17)	C16—N1—H1	110.0 (18)
C10—C9—C8	112.83 (18)	C14—N1—H1	106.1 (18)
C10—C9—H9A	109.0	C4—O1—C17	116.91 (19)
C8—C9—H9A	109.0	C8—O2—H2A	108.2 (19)
C10—C9—H9B	109.0	O3B—S1B—O1B	114.29 (16)
C8—C9—H9B	109.0	O3B—S1B—O2B	109.48 (19)
H9A—C9—H9B	107.8	O1B—S1B—O2B	112.99 (15)
C9—C10—C11	111.7 (2)	O3B—S1B—C3B	105.89 (13)
C9—C10—H10A	109.3	O1B—S1B—C3B	107.97 (10)
C11—C10—H10A	109.3	O2B—S1B—C3B	105.58 (12)
C9—C10—H10B	109.3	C6B—C1B—C2B	120.6 (3)
C11—C10—H10B	109.3	C6B—C1B—H1B	119.7
H10A—C10—H10B	107.9	C2B—C1B—H1B	119.7
C10—C11—C12	110.1 (2)	C1B—C2B—C3B	119.8 (3)
C10—C11—H11A	109.6	C1B—C2B—H2B	120.1
C12—C11—H11A	109.6	C3B—C2B—H2B	120.1
C10—C11—H11B	109.6	C4B—C3B—C2B	119.1 (2)
C12—C11—H11B	109.6	C4B—C3B—S1B	120.50 (19)
H11A—C11—H11B	108.1	C2B—C3B—S1B	120.4 (2)
C11—C12—C13	111.7 (2)	C3B—C4B—C5B	119.8 (2)
C11—C12—H12A	109.3	C3B—C4B—H4B	120.1
C13—C12—H12A	109.3	C5B—C4B—H4B	120.1
C11—C12—H12B	109.3	C6B—C5B—C4B	120.5 (3)
C13—C12—H12B	109.3	C6B—C5B—H5B	119.7
H12A—C12—H12B	107.9	C4B—C5B—H5B	119.7
C12—C13—C8	111.8 (2)	C1B—C6B—C5B	120.0 (3)
C12—C13—H13A	109.3	C1B—C6B—H6B	120.0
C8—C13—H13A	109.3	C5B—C6B—H6B	120.0
C12—C13—H13B	109.3	H1WA—O1W—H1WB	102 (3)
C8—C13—H13B	109.3		
			
C6—C1—C2—C3	−1.0 (3)	C10—C11—C12—C13	55.6 (3)
C7—C1—C2—C3	179.1 (2)	C11—C12—C13—C8	−56.5 (3)
C1—C2—C3—C4	0.4 (4)	O2—C8—C13—C12	−64.4 (2)
C2—C3—C4—O1	179.6 (2)	C9—C8—C13—C12	54.5 (2)
C2—C3—C4—C5	0.3 (4)	C7—C8—C13—C12	177.97 (18)
O1—C4—C5—C6	−179.5 (2)	C1—C7—C14—N1	−158.74 (18)
C3—C4—C5—C6	−0.3 (4)	C8—C7—C14—N1	71.9 (2)
C4—C5—C6—C1	−0.4 (4)	C7—C14—N1—C15	74.7 (2)
C2—C1—C6—C5	1.0 (3)	C7—C14—N1—C16	−160.7 (2)
C7—C1—C6—C5	−179.1 (2)	C5—C4—O1—C17	0.4 (4)
C2—C1—C7—C14	134.3 (2)	C3—C4—O1—C17	−178.9 (2)
C6—C1—C7—C14	−45.6 (3)	C6B—C1B—C2B—C3B	0.5 (4)
C2—C1—C7—C8	−97.0 (2)	C1B—C2B—C3B—C4B	−0.4 (4)
C6—C1—C7—C8	83.1 (3)	C1B—C2B—C3B—S1B	179.60 (18)
C1—C7—C8—O2	179.41 (16)	O3B—S1B—C3B—C4B	143.1 (2)
C14—C7—C8—O2	−52.9 (2)	O1B—S1B—C3B—C4B	−94.1 (2)
C1—C7—C8—C13	−63.5 (2)	O2B—S1B—C3B—C4B	27.0 (2)
C14—C7—C8—C13	64.3 (2)	O3B—S1B—C3B—C2B	−36.9 (2)
C1—C7—C8—C9	59.8 (2)	O1B—S1B—C3B—C2B	85.9 (2)
C14—C7—C8—C9	−172.50 (17)	O2B—S1B—C3B—C2B	−153.0 (2)
O2—C8—C9—C10	61.5 (2)	C2B—C3B—C4B—C5B	0.6 (4)
C13—C8—C9—C10	−54.7 (2)	S1B—C3B—C4B—C5B	−179.39 (18)
C7—C8—C9—C10	178.92 (17)	C3B—C4B—C5B—C6B	−1.0 (4)
C8—C9—C10—C11	56.0 (2)	C2B—C1B—C6B—C5B	−0.8 (4)
C9—C10—C11—C12	−55.1 (3)	C4B—C5B—C6B—C1B	1.0 (4)

### Hydrogen-bond geometry (Å, º)

Cg1 is the centroid of the C1–C6 ring.

**Table:**

*D*—H···*A*	*D*—H	H···*A*	*D*···*A*	*D*—H···*A*
O2—H2*A*···O1*W*	0.81 (3)	1.87 (3)	2.673 (3)	173 (3)
O1*W*—H1*WB*···O3*B*	0.83 (3)	1.92 (3)	2.711 (4)	159 (3)
O1*W*—H1*WA*···O2*B*^i^	0.88 (3)	1.91 (3)	2.785 (4)	175 (3)
N1—H1···O2	0.78 (3)	2.05 (3)	2.719 (2)	143 (3)
C15—H15*C*···O2*B*	0.96	2.68	3.468 (4)	140
C16—H16*A*···O1*B*^ii^	0.96	2.44	3.395 (4)	172
C2—H2···*Cg*1	0.93	3.17	3.928	140

Symmetry codes: (i) −*x*+1, −*y*+1, −*z*+1; (ii) *x*, *y*−1, *z*.
